# Application of 3D printing in the treatment of diabetic foot ulcers: current status and new insights

**DOI:** 10.3389/fbioe.2024.1475885

**Published:** 2024-11-13

**Authors:** Xinrui Li, Xin Ai, Bo Wang, Mengqian Luo, Akira Miyamoto, Mohammad Shafi Kuchay, Dechao Feng, Chi Zhang

**Affiliations:** ^1^ Department of Rehabilitation, The Affiliated Hospital of Southwest Medical University, Luzhou, China; ^2^ Department of Nishikyushu University Faculty of Rehabilitation, Fukuoka, Japan; ^3^ Division of Endocrinology and Diabetes, Medanta the Medicity Hospital, Haryana, India; ^4^ Department of Urology, Institute of Urology, West China Hospital, Sichuan University, Chengdu, China; ^5^ Division of Surgery and Interventional Science, University College London, London, United Kingdom

**Keywords:** diabetes foot ulcers, 3D printing, bio-materials, new treatments, intelligent detection

## Abstract

**Background and Aims:**

Diabetic foot ulcers (DFUs) are a serious complication of diabetes mellitus (DM), affecting around 25% of individuals with DM. Primary treatment of a DFU involves wound off-loading, surgical debridement, dressings to provide a moist wound environment, vascular assessment, and appropriate antibiotics through a multidisciplinary approach. Three-dimensional (3D) printing technology is considered an innovative tool for the management of DFUs. The utilization of 3D printing technology in the treatment of DFU involves the modernization of traditional methods and the exploration of new techniques. This review discusses recent advancements in 3D printing technology for the application of DFU care, and the development of personalized interventions for the treatment of DFUs.

**Methods:**

We searched the electronic database for the years 2019–2024. Studies related to the use of 3D printing technology in Diabetic foot were included.

**Results:**

A total of 25 identified articles based on database search and citation network analysis. After removing duplicates, 18 articles remained, and three articles that did not meet the inclusion criteria were removed after reading the title/abstract. A total of 97 relevant articles were included during the reading of references. In total, 112 articles were included.

**Conclusion:**

3D printing technology offers unparalleled advantages, particularly in the realm of personalized treatment. The amalgamation of traditional treatment methods with 3D printing has yielded favorable outcomes in decelerating the progression of DFUs and facilitating wound healing. However, there is a limited body of research regarding the utilization of 3D printing technology in the domain of DFUs.

## 1 Introduction

Nowadays, the aging of society is accelerating, and the growth of age and the degradation of physiological functions will induce a variety of diseases, such as various cancers and chronic diseases (DeSantis et al., 2019; Feng et al., 2024; Shen et al., 2022; Feng DC et al., 2023). Diabetes mellitus (DM) is the most common chronic metabolic disease with high incidence, which brings serious public health burden (Collier et al., 2024; Lin et al., 2023). The global prevalence of DM is estimated at 9.3 percent (463 million people) in 2019. By 2030 and 2045, this proportion may increase to 10.2% (578 million) and 10.9% (700 million) respectively (Magliano et al., 2019).

Diabetic foot (DF) is one of the serious complications of diabetes (Du et al., 2023; Bellomo et al., 2022). Eighty five percent of individuals with diabetes mellitus undergoing lower extremity amputation have had DFUs (Thorud et al., 2016; Lepäntalo et al., 2011). Therefore, early identification, prevention and effective DF management are essential to improve the quality of life of DM patients (Guo et al., 2024). DF is currently managed primarily through medication, wound care, and surgery. Among these treatments, management based on 3D printing has gained the attention of researchers.

3D printing technology facilitates the production of personalized equipment with complex structures, and also offers new possibilities for customized solutions (Dong NJ et al., 2022; Silva et al., 2022; Chen et al., 2024; Wu et al., 2024; Jiang et al., 2024). In diabetic foot management, 3D printing can customize wound dressings and assistive devices, and can be combined with biomaterials to promote wound healing and functional restoration of the foot (Beach et al., 2021; Armstrong et al., 2022; Collings et al., 2021; Jørgensen et al., 2018; Wang et al., 2023). We provide a comprehensive review of the implementation of 3D printing technology in the integrated management of DFUs.

## 2 Methodology

### 2.1 Search strategy

The literature search was conducted in electronic databases for the years 2019–2023. The search strategy in PubMed was as follows: ((“Diabetic Foot”[Mesh]) OR (Diabetic foot ulcer[Title/Abstract])) AND ((“Printing, Three-Dimensional”[Mesh]) OR ((((((((((((((((((((3D printing[Title/Abstract]) OR (Printing, Three Dimensional[Title/Abstract])) OR (Printings, Three Dimensional[Title/Abstract])) OR (Three-Dimensional Printings[Title/Abstract])) OR (3-Dimensional Printing[Title/Abstract])) OR (3 Dimensional Printing[Title/Abstract])) OR (3-Dimensional Printings[Title/Abstract])) OR (3-Dimensional Printings[Title/Abstract])) OR (Printings, 3-Dimensional[Title/Abstract])) OR (3-D Printing[Title/Abstract])) OR (3 D Printing[Title/Abstract])) OR (3-D Printings[Title/Abstract])) OR (Printing, 3-D[Title/Abstract])) OR (Printings, 3-D[Title/Abstract])) OR (Three-Dimensional Printing[Title/Abstract])) OR (Three Dimensional Printing[Title/Abstract])) OR (3D Printing[Title/Abstract])) OR (3D Printings[Title/Abstract])) OR (Printing, 3D[Title/Abstract])) OR (Printings, 3D[Title/Abstract]))). In addition, the reference lists of the included articles were investigated to identify other relevant articles that could not be found through the initial electronic search strategy.

### 2.2 Inclusion and exclusion criteria

Studies related to the use of 3D printing technology in DF were included. Studies, review papers, book chapters, conference abstracts, reviews and research protocols published in any language other than English were excluded. The first step was to eliminate duplicate articles by looking at titles and abstracts through EndNote. Next, titles were screened to remove irrelevant articles. Then, abstracts and full texts of relevant articles were read and screened for inclusion based on predefined criteria. Finally, criteria-compliant articles were included.

### 2.3 Result

**FIGURE 1:**
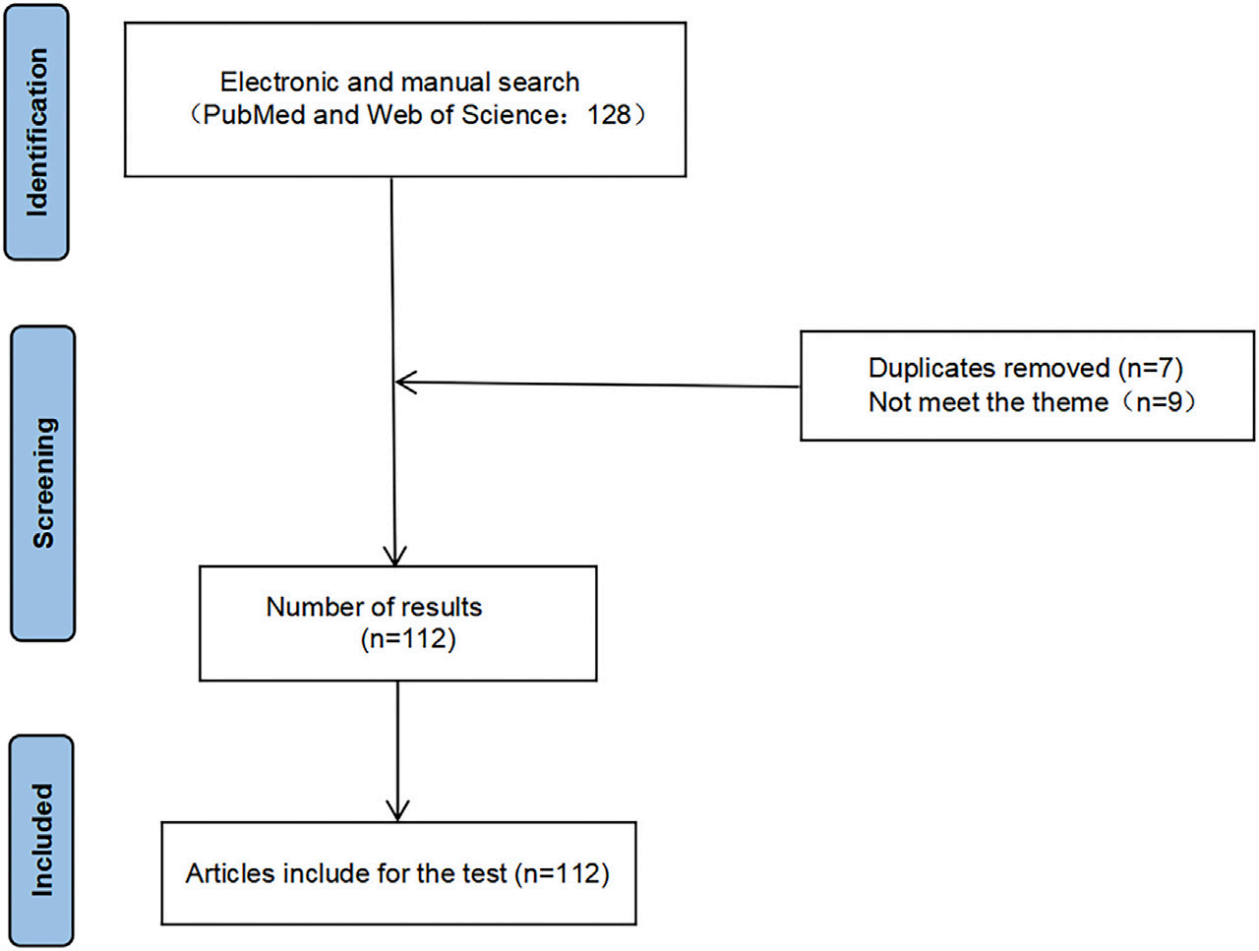
Flow chart of literature search.

A total of 25 identified articles based on database search and citation network analysis. After removing duplicates, 18 articles remained, and three articles that did not meet the inclusion criteria were removed after reading the title/abstract. A total of 97 relevant articles were included during the reading of references. In total, 112 articles were included. The flow chart of this study was presented in Figure 1.

## 3 Models

### 3.1 3D printing of DFUs models

The paucity of drugs for DFU treatment in the clinic is partly due to the lack of good experimental models to predict their effects. The mouse model is superior to the human body in terms of wound repair due to the abundance of hair follicle stem cells and growth factors, leading to the questioning of the mouse-based diabetes model (Phang et al., 2021). The 3D printed of DFUs model resembles human skin in anatomical structure, mechanical and biochemical features, and transcriptomics and proteomics also show similarities to human skin development (Admane et al., 2019). The analysis of the 3D models is presented in Table 1.

**TABLE 1:** Analysis of the 3D models.

Study	Objective	Evaluated parameter(s)	Main conclusion	Research direction
Tan et al., 2022	Evaluate the efficacy of 3D printing for the treatment of severe skin wounds	Conducting dialectical summaries	3D printed scaffolds is an effective approach to managing cutaneous wound healing	3D model
Phang et al., 2021	Study of different *in vitro* 3D skin models and 3D angiogenesis models	Feasibility and applicability of 3D model	Bio-3D printing and skin microarray models as diabetic wound models has good research prospects	3D model
Vu et al., 2021	Scaffold-based and scaffold-free 3D cell culture systems that mimic *in vitro* environments	Construct Biological Scaffolds and analysis of ECM protein regulation	Scaffold-free system suit for analysing ECM protein regulation	3D model
Smith et al., 2021	Surveyor present a 3D HSE model	Cellular analysis and assessment	3D HSE model is used to study macrophage-related inflammation in diabetes and as a drug testing tool to evaluate new treatments for the disease	3D model
Singh et al., 2020	*In situ* bioprinting *versus* conventional printing	The need and utility for *in situ* bioprinting	*In situ* bioprinting may be favored when tissues are to be fabricated or repaired directly on the intended anatomical location in the living body	3D model
Smith et al., 2020	Importance of human-derived ECM for constructing 3D skin models	controlled trial	This humanized skin-like tissue decreases dependency on animal-derived ECM while increasing cellular complexity can enable screening inflammatory responses in tissue models of human skin	3D model
Lin et al., 2019	To investigate the expression of miR-217 and HIF-1α/VEGF pathway in patients with diabetic foot ulcer and its effect on angiogenesis in DFUs rats	Animal experiments, genetic testing and pathway analysis	Inhibiting miR-217 could upregulate HIF-1α/VEGF pathway to promote angiogenesis and ameliorate inflammation of DFU rats, thereby effectively advancing the healing of ulcerated area	3D model
Admane et al., 2019	Application of 3D cell culture system to diabetic diseases	Feasibility and applicability of 3D system	3D models offer a advantage in obtaining physiologically relevant information	3D model
Sun et al., 2018	A case evaluates the safety and effectiveness of 3D-printed scaffold in chronic wounds	controlled trial	3D-printed scaffold was convenient to use, have the potential to improve wound healing rates and provided a safe and effective way for treating chronic wounds	3D model
Intini et al., 2018	The fabrication of porous 3D printed chitosan scaffolds for skin tissue regeneration and their behavior in terms of biocompatibility and toxicity toward human fibroblasts and keratinocytes	Cellular experiments with co-staining and other assays	3D printed scaffolds improve the quality of the restored tissue with respect to both commercial patch and spontaneous healing	3D model

^a^ 3D: Three-dimensional.

^b^ ECM: extracellular matrix.

^c^ HSE: human skin equivalent.

^d^ DFU: diabetic foot ulcer.

### 3.2 3D organotypic skin models

3D printing-based skin models can accelerate wound healing by reducing inflammation, inhibiting fibrosis or increasing angiogenesis or regeneration (Kondej et al., 2024).3D skin modelling is divided into scaffold and scaffold-free systems, with scaffold modelling being widely used due to altered porosity, surface chemistry and permeability. Scaffolds composed of biopolymers mimic the extracellular matrix (ECM) and provide support and signals to cells, creating organotypic models that mimic native human skin (Phang et al., 2022). Cross-linked polymer hydrogels and matrix gels are commonly used scaffold materials, in addition to nanofibers, collagen sponges, agarose peptide microgels, polystyrene and polycaprolactone (Mohandas et al., 2023). Stent materials function differently and are used to replicate disease outcomes. 3D printed scaffolds are convenient, support high cellular loads and remain viable to accelerate healing, and also deliver stable antibiotics for effective treatment of chronic wounds (Sun et al., 2018; Glover et al., 2023). Intini et al. fabricated chitosan (CH) porous 3D printed scaffolds for skin regeneration. They loaded normal human dermal fibroblasts and keratin-forming cells into the scaffold holes to form a skin-like layer. The CH scaffolds promoted wound healing in diabetic rats compared to commercial patches and self-healing (Intini et al., 2018). Furthermore, the scaffold-free system serves as a scaffold alternative structure that alleviates poor biocompatibility. Such systems are essential for analyzing ECM protein regulation in human skin and can transfer cells from two to three dimensions, inducing deep upregulation of matrix body proteins and generating complex tissue-like ECM (Vu et al., 2021). Engineered skin substitutes cannot fully mimic the complex native environment of wound healing. However, 3D printed scaffolds offer a solution to the recurrent problem of limited donor tissues and high donor site morbidity seen in tissue transplantation, while *in vitro* their fragile structure may lead to tissue damage, and *in vitro* bioprinting and artificial implantation carry the risk of contamination (Tan et al., 2022; Singh et al., 2020).

### 3.3 3D hyperglycemic wound models

Fibroblast growth factor (FGF) is an important factor to consider in the design of DFU models. 3D human skin equivalent (HSE) models consisting of cells from DFU patients can induce an inflammatory response and have been used to study diabetic inflammation, drug testing, and to reduce reliance on animal-derived ECM (Smith et al., 2021; Smith et al., 2020). The researchers also developed a three-dimensional hyperglycemic wound model of normal human keratinocytes, demonstrating common phenotypes of DFU such as re-epitheliazation, granulation and damage caused by keratinocyte over-proliferation (Phang et al., 2021). Induced pluripotent stem cells (iPSCs) were generated from fibroblasts of patients with diabetes and from fibroblasts of healthy persons harvested from skim. They were modified and induced to differentiated into fibroblasts. Research has found that the gene expression and characteristic matrix composition exhibited by iPSC-derived fibroblasts in 3D dermal-like tissues were similar to those of primary fibroblasts, and they continuously promoted matrix remodeling and wound healing in chronic wound environments, demonstrating therapeutic potential. IPSC reprogramming is considered effective in promoting cell healing to eliminate genetic traits (Pastar et al., 2021; Kashpur et al., 2019). Currently, there are still challenges in printing biological models in high permeability and high glucose environments. Based on existing studies progress, we need to develop more mature DF trauma models.

### 3.4 3D angiogenesis model

**FIGURE 2:**
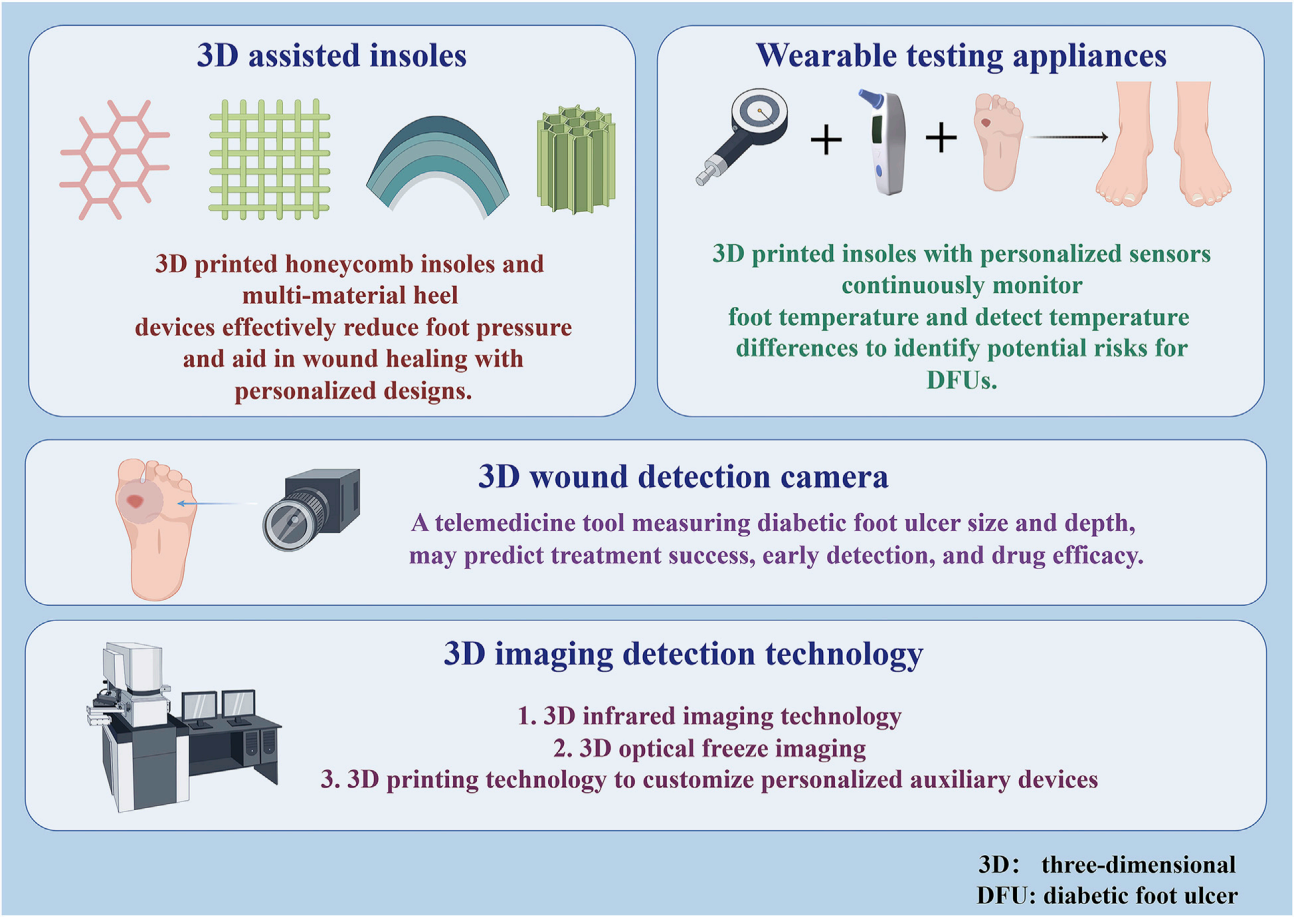
3D printing of experiment models.

DF vascular injury and impaired healing of diabetic ulcers are associated with poor angiogenesis of granulation tissue (Lin et al., 2019). Hyperglycemia induced increased production of reactive oxygen species and the exacerbation of apoptosis during ischemia (Han et al., 2021) which it is also considered an influential factor in the injury of DFUs. The 3D endothelial cell germination test is more reflective of the angiogenic process of endothelial cells than the traditional 2D test and can be used as a screening tool for hyperglycemic applications (Phang et al., 2021). 3D printed endothelial progenitor cell skin patches together with adipose-derived stem cells accelerate wound closure, re-epithelialization, neovascularization and blood flow (Kim et al., 2018). 3D printing not only significantly improves the flexibility and precision of *in vitro* modelling, but also dramatically reduces costs and shortens development cycles through high customisation, rapid fabrication of complex structures, and the use of a wide range of biocompatible materials. This technology facilitates interdisciplinary integration and strengthens collaboration between biology, medicine and engineering, making it a key tool to support innovation in multiple fields such as clinical research, drug screening and disease modelling. The application of joint 3D printing technology to manufacture experimental models is presented in Figure 2.

## 4 Combined 3D-printed therapies

### 4.1 Autologous minimal manipulation of homologous adipose tissue (AMHAT)

Adipose tissue contains high levels of cell growth factors that can promote angiogenesis and wound remodeling (Lavery et al., 2007). Furthermore, anti-inflammatory cytokines and healing-related peptides may positively affect wound healing (Pallua et al., 2009). Thus, autologous micro-fragmented adipose tissue can significantly improve the healing of small amputations following DFU surgery (Lonardi et al., 2019). For patients, subcutaneous liposuction to extract fat cells is relatively simple and less painful (Gimble et al., 2007). In a single-arm pilot study, ten patients with chronic DFUs were treated with autologous minimally manipulated homologous adipose tissue (AMHAT). During follow-up, the patient wounds healed well (Armstrong et al., 2022). 3D printing was combined with minimally manipulated ECM (MA-ECM) to create bio scaffolds that increase the speed of wound healing in patients (Kesavan et al., 2021; Kesavan et al., 2024; Yoon and Song, 2024). Fibrin gel is biocompatible and mimics the clotting process, reduces inflammation, and promotes cell adhesion and proliferation to accelerate healing. In addition, it has good mechanical strength. Thus, Fibrin gel was added to a 3D-AMHAT scaffolds, which not only promote wound healing, were easily absorbed without interfering with the healing process, but were also strong enough to withstand changes in mechanical stress during the healing process, thus further accelerating wound healing (Bajuri et al., 2023). By combining autologous adipose tissue and 3D printing technology to create a biocompatible and mechanically robust scaffold, the effectiveness and speed of diabetic foot wound healing can be significantly improved. 3D printing combined with autologous fat grafting helps DF wounds healing and is considered a new approach to treatment at DFUs.

### 4.2 Combined 3D printed guide-guided lateral tibial transport

Transverse tibial bone transfer (TTBT) is a novel surgical approach to treat DFUs, and several clinical trials have confirmed its efficacy (Godoy-Santos et al., 2017; Kallio et al., 2015). However, during conventional bone transfer, the periosteum may be damaged, and deviations in the angle of screw placement can result in the direction of bone transfer that is not perpendicular to the slice, which can lead to postoperative spatial heterogeneity on both sides of the body and affect the growth of the microvascular network (Randon et al., 2010). Yuan-Wei Zhang et al. performed TTBT under the guidance of a 3D-printed guide plate with DM patients. This new technique is effective in preserving the relative integrity of the bone window and periosteum. Moreover, surgeons can simulate the surgical plan on a 3D model, improving the accuracy of the operation (Wang et al., 2023).

### 4.3 Functional dressings in conjunction with 3D printing

**FIGURE 3:**
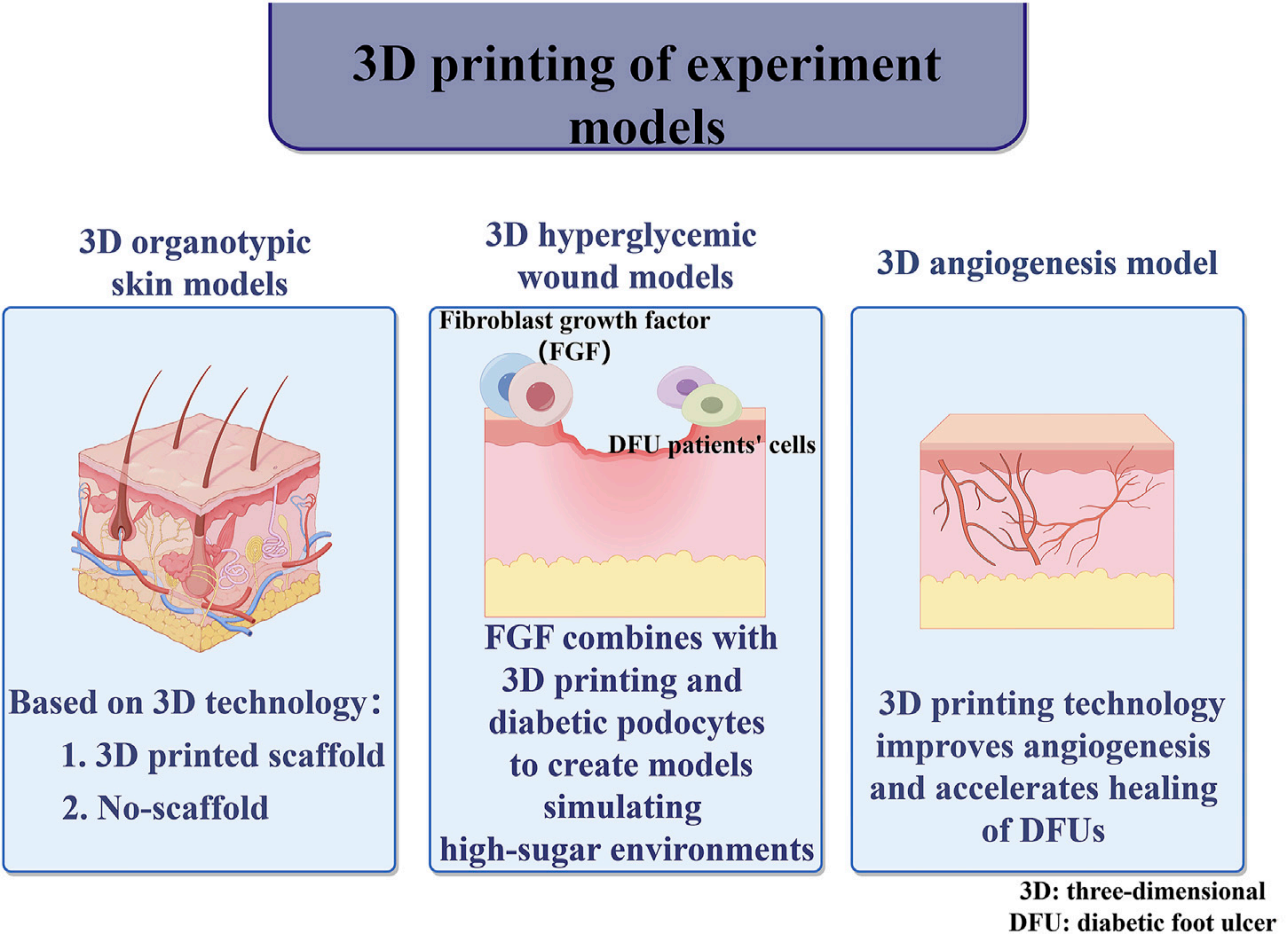
Combined 3D-printed therapies.

DFU is characterized by persistent chronic inflammation, granulation tissue formation, and reduced vascularization (Hu and Xu, 2020). Single dressings have limited effect, DFU dressings are enhanced with bioactive molecules. Hydrogels are considered to be excellent wound dressings (Tran et al., 2023; Feng et al., 2023; Sarkar and Poundarik, 2022; Glover et al., 2021; Gomes et al., 2020). Multifunctional bioprinter dressing increase the thickness of wound granulation tissue and promote the formation of blood vessels, hair follicles and collagen fiber networks (Huang et al., 2022). For examples: The addition of VEGF to 3D-printed dressings enhanced the proliferation of endothelial cells, promoted the formation of blood vessels in the body, and accelerated wound healing. Interleukin four and antioxidant-rich autologous bio gel protected fibroblasts in patients with diabetic foot ulcers (DFUs) and facilitated wound healing (Tan et al., 2020; Mashkova et al., 2019; Yang et al., 2022). In another development, a silver vinyl-based 3D-printed antimicrobial ultra-porous polyacrylamide (PAM)/hydroxypropyl methylcellulose (HPMC) hydrogel dressing was designed with a porosity of 91.4%. It featured open channels that allowed it to absorb water rapidly, taking in up to 14 times its own weight. The large pores helped reduce swelling, minimized the risk of dressing dislodgment, and promoted the healing of infected wounds (Liu et al., 2021). Furthermore, 3D-printed silver gelatin dressings demonstrated good antimicrobial properties and promoted wound healing. A hydrogel infused with nanofibers was used to synthesize tissue-like structures and was applied to rat skin breaks, showing excellent biocompatibility and antibacterial effects (Bahmad et al., 2021; Jin et al., 2023; Wan et al., 2019). Additionally, the encapsulation of antimicrobial peptides and PDGF-BB into porous 3D radially aligned nanofiber scaffolds (RAS) allowed for the recruitment of fibroblasts, endothelial cells, and keratinocytes to clear bacterial infections and enhance granulation tissue formation (Li et al., 2024). The researchers also undertook a technological update to develop a coaxial microfluidic 3D bioprinting technology combining flow-assisted dynamic physical crosslinking and calcium ion chemical dual crosslinking method, designing a biologically active multilayer core-shell fibrous hydrogel loaded with PRP. The prepared hydrogel exhibited excellent water absorption and retention capabilities, good biocompatibility, and broad-spectrum antibacterial effects (Huang et al., 2023). The combination of 3D printing technology and biomaterials such as cytokines enables wound dressings to be personalized, with precise control of the material structure, multifunctional, and effective in promoting angiogenesis, tissue regeneration and optimizing drug release. This significantly improves wound healing efficiency and therapeutic efficacy. The analysis of the 3D-printed therapies is presented in Table 2. And The application of 3D printing technology combined with biological materials in surgery and wound care was presented in Figure 3.

**TABLE 2:** Analysis of the 3D-printed therapies.

Study	Objective	Evaluated parameter(s)	Main conclusion	Research direction
Bajuri et al., 2023	To determine the efficacy of 3D-bioprinted autologous adipose tissue grafts on DFUs	Post-intervention regular observation of wounds in diabetic patients	Autologous adipose tissue grafting using 3D bioprinter promotes wound healing with high-quality skin reconstruction	3D-printed therapies
Tran et al., 2023	Assessing the role of nanomaterials and other biomaterials in wound healing	Feasibility and applicability of new materials	Novel Biomaterial Prevents/Treats Infections, accelerates wound healing and monitors wound healing status	3D-printed therapies
Jin et al., 2023	To explore the effects of 3D bioprinting methacrylate gelatin hydrogel loaded with nano silver on full-thickness skin defect wounds in rats	Animal experiments one-way analysis of variance Bonfroni correction and independent samples t-tests were used to statistically analyse the data	Silver-containing methacrylate gelatin hydrogel has good biocompatibility and antibacterial properties. Its 3D bioprinted double-layer structure can better integrate with new formed tissue in the skin defect wounds in rats and promote wound healing	3D-printed therapies
Huang et al., 2023	Therapeutic efficacy Platelet-rich plasma-loaded bioactive multi-layer shell-core fibrous hydrogels	Frequency of administrationwound healing rate angiogenesis rate analysis	The bioactive fibre hydrogel effectively reduces inflammation, promotes granulation tissue growth and angiogenesis, facilitates the formation of high-density hair follicles, and generates a regular network of high-density collagen fibres	3D-printed therapies
Tian et al., 2024	Providing a new solution for manufacturing personalised DF insoles	A three-step protocol for the development and evaluation of this therapeutic footwear	The involvement of end-users (diabetic patients) will enable the definition of user requirements and contexts of use to develop design solutions for the footwear	3D-printed therapies
Armstrong et al., 2022	AMHAT therapies to support good quality basic care	Clinical trials with timed observation of wounds	Treatment of bioprinted AMHAT appears to be a safe and potentially effective treatment modality for patients with chronic DFUs	3D-printed therapies
Huang et al., 2022	Evaluation of the effectiveness of multifunctional medical dressings	Inflammation analysis and wound healing analysis	The multifunctional 3D dressing reduced inflammation, effectively increased the post-healing thickness of granulation tissue, and promoted the formation of blood vessels, hair follicles and highly oriented collagen fiber networks	3D-printed therapies
Bahmad et al., 2021	Find alternatives to traditional treatments with 3D printed therapies	Summary analysis of studies related to database searching	Nanofiber-skin substitutes hold promise for treatment of patients suffering from DFUs and inspire novel strategies that could be applied to other organ systems as well	3D-printed therapies
Kesavan et al., 2021	The efficacy of MA-ECM prepared from autologous homologous adipose tissue by using 3D bioprinting in DFUs	Reduction of wound size and the appearance of epithelialization were evaluated	MA-ECM-based treatment accelerates wound healing	3D-printed therapies
Tan et al., 2020	Improvement and development of effective dfu-specific wound dressings and treatments	Summary analysis of studies related to database searching	Co-development of 3D bioprinting technologies with novel treatment approaches to mitigate DFUs -specific pathophysiological challenges will be key to limiting the healthcare burden associated with the increasing prevalence of DM	3D-printed therapies
Hu and Xu, 2020	Efficacy based on polysaccharide hydrogels	Summary analysis of studies related to database searching	Polysaccharide-based hydrogels can provide suitable moisture for the wound and act as a shield against bacteria	3D-printed therapies
Gomes et al., 2020	The efficacy of dual antimicrobial peptide	locally delivered into the model	The local application of the dual-antimicrobial peptides biogel constitutes a potential complementary therapy for the treatment of infected DFUs	3D-printed therapies
Mashkova et al., 2019	3D skin printing mimics the effects of native wound environments	Summary analysis of studies related to database searching	3D-bioprinting plays a vital role in developing a complex skin tissue structure for tissue replacement approach in future precision medicine	3D-printed therapies
Lonardi et al., 2019	Injection of autologous microfragment adipose tissue compared with standard treatment	Assessment of wound healing in terms of safety, feasibility technical success, recurrence rate, skin deviation and pain intensity	The local injection of autologous micro-fragmented adipose tissue is a safe and valid therapeutic option able to improve healing rate following minor amputations of irreversible DFUs	3D-printed therapies

^a^ 3D: Three-dimensional.

_b_ AMHAT: autologous minimal manipulation of homologous adipose tissue.

^c^ MA-ECM: minimally manipulated autologous extracellular matrix.

^d^ DFU: diabetic foot ulcer.

^e^ DF: diabetic foot.

^f^ DM: diabetes mellifluous.

## 5 Auxiliary tools for joint 3D printing

### 5.1 3D assisted insoles

3D printing-assisted customized insoles can reduce the incidence of DFUs and reduce plantar pressure, thereby mitigating the risk of DFUs and infections (Leung et al., 2022). Below we present the application of 3D printing-assisted manufacturing of insoles in diabetic foot management.

#### 5.1.1 Auxiliary materials

The 3D printed circular honeycomb structure insole can withstand large deformations, improve energy absorption and breathability, and adapt to different foot shapes. After finite element analysis, the addition of a hemispherical heel pad effectively reduces contact force and pressure (Leung et al., 2022). Personalized metamaterials have the characteristics of the substrate and the lattice microstructure inside. The features can meet individual needs and reduce plantar stress (Muir et al., 2022). Pressure-reducing shoes are thought to impair balance, but researchers have developed a 3D-printed rocker midsole and self-adjusting insole that can reduce plantar pressure and maintain balance (Malki et al., 2024).

#### 5.1.2 Graded unloading pressure

Pressure unloading is a method of relieving pressure on the foot and promoting wound healing. There are several offloading devices, such as walkers, half-shoes, orthotics, felt foam and total contact casts (TCC) (Nabuurs-Franssen et al., 2005). TCC has the disadvantage of continuous irritation of the skin and skin ulcers on the plaster and a risk of muscle atrophy (Armstrong and Lavery, 1998; Caravaggi et al., 2000). Felt and foam conditioning dressings applied over and proximal to ulcers and applied to the foot are more effective (Meneses et al., 2020). Felt and foam dressings are not as effective at reducing pressure as casts, walkers or half-shoes. Walkers and half-shoes, although convenient and inexpensive, are not as effective at reducing pressure as TCC (Lavery et al., 1996). For neurogenic and neurochemical foot ulcers patients, increased biomechanical stress is one of the most important ways leading to ulceration (Nabuurs-Franssen et al., 2005). Localized generation of high loads in the soft tissues at the site of stress concentration can lead to cell and tissue damage. This, in turn, increases the risk of secondary ulceration in these areas (i.e., where the insole material passes between the insole and the holes) (Shaulian et al., 2023). The Graded-Stiffness (GS) method is a novel unloading solution that combines 3D printed polygonal heels and stiffness distribution designed to redistribute plantar pressure to prevent and treat heel ulcers. The structure is progressively stiffened from the inside to the outside, optimizing the unloading position through graded stiffness and material properties. Finite element analysis shows that this multi-material device effectively reduces heel pressure and distributes stress (Shaulian et al., 2023; Shaulian et al., 2022). The optimal stiffness of the sole is correlated with the user’s body weight (BMI), in order to minimize foot pressure (Chatzistergos et al., 2020). Combining mechanical with kinematic measurements can better detect plantar loading in specific foot regions (Giacomozzi et al., 2014). The cone-beam computed tomography (CBCT) was employed to generate a 3D skeletal model of the foot. They performed image segmentation and conducted precise angular measurements in various anatomical planes. The objective was to establish a relationship between bone structure and plantar loading (Belvedere et al., 2020). The analysis of the auxiliary tools is presented in Table 3.

**TABLE 3:** Analysis of the auxiliary tools.

Study	Objective	Evaluated parameter(s)	Main conclusion	Research direction
Shaulian et al., 2023	Development of insoles to distribute plantar pressure	A novel offloading method	The precise selection of graded stiffness unloading helps to create personalized insoles	Auxiliary tools
Leung et al., 2022	Pressure reducting insoles in the DF	Re-entrant structure internal angle to peak contact force, average heel pressure and heel to gasket contact surface integral allowances	The honeycomb structure relieves pressure on the foot and reduces the occurrence of ulcers and wounds exacerbation	Auxiliary tools
López-Moral et al., 2022	Validate a novel 3D foot scanner app for selecting the proper fitting therapeutic footwear	Foot skeletal muscle analysis and foot scan analysis	Enables deeper profiling of the DF	Auxiliary tools
Muir et al., 2022	Advantages of personalised materials for 3D printing	Analysis of maximum peak plantar pressure and time to pressure in different states	The ability to manufacture the 3D printed personalized metamaterials insoles and demonstrates their ability to reduce plantar pressure	Auxiliary tools
Lasschuit et al., 2021	3D wound cameras	Evaluating the accuracy of the device in terms of wound depth, width and shape size	3D wound cameras provide a more comprehensive understanding of the ulcer to improve subsequent outcomes	Auxiliary tools
Belvedere et al., 2020	Analysis of Skeletal Muscle by Wearable 3D Printing Devices in DFUs	Analysis of dynamic temperature parameters and rate of temperature change	Temperature rise time measured at the plantar surface may be indicative biomarker for differences in biomechanics and vascularisation	Auxiliary tools
Malone et al., 2020	3D wound imaging	Intraclass correlation coefficient assessment, linear regression and pearson correlation coefficient test	3D wound imaging could be effective prognostic markers to wounds progression to healing and closure. It provide important early identification of wounds	Auxiliary tools
Jørgensen et al., 2018	3D-WAM camera	Wound size, shape and depth analysis 2D,3D image analysis	the 3D-WAM camera is an accurate and reliable method which is useful for several types of wounds	Auxiliary tools
van Doremalen et al., 2020	Infrared 3D thermography	Concept certification analysis of infrared imaging 3D maps	3D thermal foot images inform assessment of 3D skin temperature in the DF	Auxiliary tools

^a^ 3D: Three-dimensiona.

^b^ DF: diabetic foot.

^c^ DFU: diabetic foot ulcer.

### 5.2 Wearable testing appliances

By measuring the temperature difference between the same parts of the feet, we can find the potential risk of DFUs and the common alert threshold is 2.2°C (Lavery et al., 2004). Patients doing self-care at home are often unable to accurately self-assess their condition, and are often treated too late when their feet become necrotic. The researchers used foot sensors to measure differences in skin temperature, which alerts when the temperature reaches an alarm threshold, and the data can be displayed, stored, or transmitted. However, existing devices are unable to study the complex dynamics of temperature changes over time (Martín-Vaquero et al., 2019). Recent studies have used 3D printed insoles equipped with personalized anatomical sensors to continuously monitor foot temperature, taking into account individual differences. A study showed that foot temperatures rose significantly faster in diabetic patients than in controls in the sitting position, at the bunion and at the head of the fifth metatarsal. This was the first time that foot temperature changes between two groups was quantified and may reveal new biomarkers associated with differences in soft tissue and angiogenesis (Beach et al., 2021). Furthermore, sensor-based insoles should also consider humidity parameters when detecting pressure and temperature (Tian et al., 2024). In another study, the multifunctional Janus membrane (3D chitosan sponge-ZE/polycaprolactone nanofibers-ZP) is thought to monitor and treat diabetic wounds, with its unidirectional water transport and strong antimicrobial capacity aiding wound healing. The membrane also monitors wound status through color and fluorescence changes, providing a basis for early intervention in diabetic patients (Liu et al., 2024).

### 5.3 3D wound detection camera

According to international guidelines, effective assessment of the size and depth of diabetic foot ulcers improves treatment success. Current assessment methods include the use of disposable rulers and metal probes (Monteiro-Soares et al., 2020). However, there are several drawbacks, including subjective error, variability in measurement time, horizontally transmitted infections and clinical waste (Fernández-Torres et al., 2020). In response to changes in ulcer size, the researchers developed the WAM 3D monitoring camera for wound assessment, which is not limited by wound size, is particularly effective in measuring heels, toes, and curved areas of the body, and is a non-invasive means of reducing the risk of infection, with digitized images suitable for telemedicine applications (Jørgensen et al., 2018; Rasmussen et al., 2015). A study analyzed 63 ulcers in 38 diabetic foot patients and found that a 3D camera effectively measured the ulcerated area and that the measurements correlated linearly with healing time, which could be used as a prognostic marker and an early identification criterion, as well as an indicator of medication efficacy (Lasschuit et al., 2021; Malone et al., 2020; Vangaveti et al., 2022). However, we still need new experiments to establish this modality.

### 5.4 3D imaging detection technology

**FIGURE 4:**
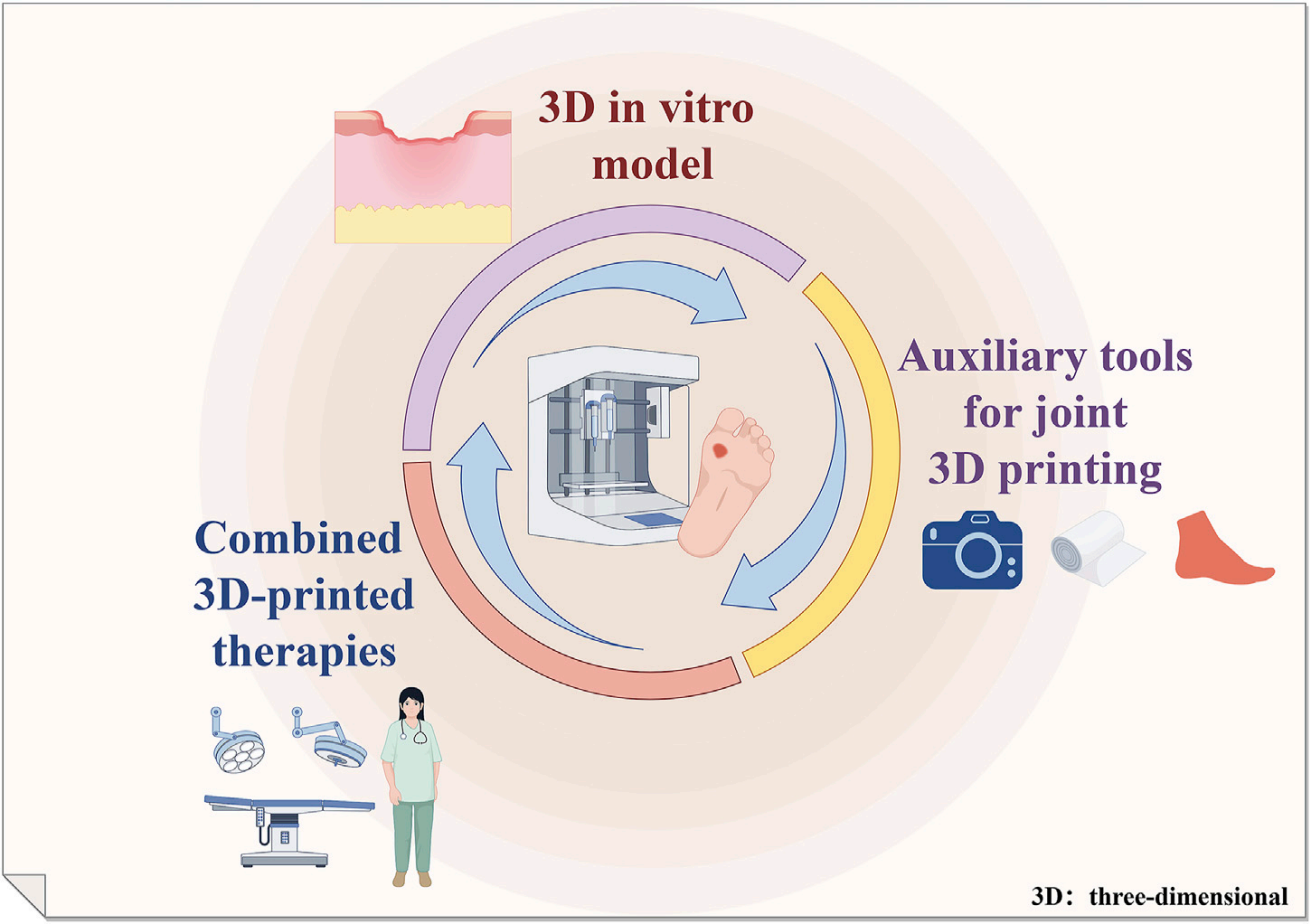
Auxiliary tools for joint 3D printing.

Thermal imaging technology provides early warning and prevention of DFU by monitoring temperature changes in the feet of diabetic patients. However, commonly used infrared cameras can only capture 2D images, requiring multiple shots to obtain a complete ulcer view. Such operation is not feasible in an already busy clinical practice and at home (van Doremalen et al., 2020; Liu et al., 2015; Rismayanti et al., 2022). Therefore, the researchers used 3D infrared imaging to overcome the shortcomings of two-dimensional imaging. The technique clearly shows the temperature difference between ulcers and normal tissue, identifies potential ulcers and danger zones, and also detects diffuse temperature elevation, suggesting inflammation or infection. It is able to analyze information about ulcers and differentiate between background and normal tissue, and the camera’s viewing angle and distance have a low impact on imaging (Liu et al., 2015). 3D optical cryo-imaging was used to assess the redox state of DFU wounds. In experiments, wound redox status in diabetic mice was quantified by *in vivo* fluorescence and 3D optical cryo-imaging and found to be correlated with mitochondrial dysfunction and increased oxidative stress, as well as the wound size. This technique can be used as a non-invasive indicator to assess complex wound healing (Mehrvar et al., 2019). Specific benefits of 3D printing technology in the manufacture of assistive devices include: individualized customization for patients, providing unparalleled comfort and functionality; orthopedic appliances designed to better fit the patient’s bone and muscle structure, reducing pressure points and enhancing orthopedic performance; rehabilitation devices such as gait trainers, which are customized to incorporate biomechanical modelling to improve the efficiency of rehabilitation; and sports aids such as knee pads and insoles, which reduce the risk of sports injuries and enhance sports performance through precise fit. In addition, 3D printing can also facilitate the upgrading and updating of diabetic foot testing equipment. These innovations not only enhance the functionality and user experience of assistive devices, but also promote the popularity and cost-effectiveness of personalized healthcare services. The application of 3D printing technology in assistive devices and ulcer monitoring is shown in Figure 4.

## 6 Material diversity in 3D bioprinting

3D bioprinting creates 3D functioning tissues/organs by precisely depositing bioink made of matrix, biological components and living cells (Xu et al., 2022). 3D printed products combined with biomaterials could control the flexible release of drugs or factors and protect sensitive biomaterials from the harsh wound environment to ensure treatment effectiveness (Pop and Almquist, 2017). Below I will introduce several common bioprinting materials. Hydrogels, owing to their excellent biocompatibility, biodegradability, and ability to mimic the extracellular matrix, are crucial in bioprinting. They support cell adhesion, growth, and crosslinking, with controllable degradation rates, making them ideal materials for promoting cell proliferation and tissue regeneration (Wang et al., 2022; Mandrycky et al., 2016). Natural materials such as gelatin and fibrin-based materials have good biocompatibility in 3D printing and can enhance cell function. For bioinks without intrinsic binding sites, incorporating cell-binding peptides (such as RGD sequences) could improve cell adhesion and viability (Cadena et al., 2021; Cadamuro et al., 2023). Biopolymers, such as gelatin methacryloyl (gelMA), chitosan, and hyaluronic acid, were advantageous because they mimicked the properties of natural ECM, had low immunogenicity, and could be modified to include motifs in their chemical structure to promote cell activity (Yue et al., 2015). Nanofibers are sustainably renewable, non-toxic, have a high specific surface area and aspect ratio and excellent mechanical properties (Li et al., 2021). Moreover, nanomaterial-based hydrogels have strong rheological properties, processability and electrical stimulation responsiveness, and also promote tissue regeneration (Tang et al., 2018; Hasan et al., 2018). Biomaterials are widely researched for their unique and superior properties. Different tissue-specific biomaterials containing cytokines and immunomodulatory properties encouraging tissue regeneration have been designed and implanted into locations of injured tissue to increase the therapeutic effectiveness of tissue regeneration (Xiong et al., 2022). Despite the great potential of bioprinting technology, there are deficiencies in biomaterials, such as insufficient mechanical strength of bioinks and challenges in precisely controlling the degradation rates of hydrogels. Additionally, some materials may be incompatible with bioprinting technology, leading to issues like clogging and reduced printing accuracy. The porosity of natural materials also limits cellular penetration and tissue integration. Consequently, the design and optimization of biomaterials still require further in-depth research (Heinrich et al., 2019). In summary, biomaterials have significant potential for development in the future.

## 7 Where is 3D printing going?

DM is a serious health problem that cannot be cured although existing drugs can alleviate the symptoms there is an urgent need to gain a deeper understanding of this pathology and to develop new models of the disease. Organoid technology offers an important opportunity to accurately mimic *in vivo* tissues by building 3D structures (Tsakmaki et al., 2020). 3D technology accelerates skin tissue regeneration and wound healing by accurately mimicking the physiological microenvironment and enhancing the complex network of inter-cellular interactions and bio-signal transduction, which allows the cells that promote wound repair to exhibit higher levels of viability and differentiation (Choudhury et al., 2024). Current 3D skin models, although partially successful in clinical applications, still have limitations due to the lack of elements such as immune cells, blood vessels, nerves and sweat glands. Secondly, there is a growing demand from patients and physicians for improved skin sensation and regeneration. Creating a unified bioink model of the skin that incorporates all these elements remains a major challenge (Ansaf et al., 2023; Tao et al., 2019). Topical treatment with dressings as part of DFU management practices creates a protective physical barrier, maintains a moist environment, and drains exudate from DFU wounds (Jiang et al., 2023). However, there are fewer types of clinically applied dressings, which need to be changed frequently, consume a lot of manpower and financial resources, and are ineffective, affecting the confidence of doctors and patients. At the same time, the high price of dressings also reduces patient compliance (Jiang et al., 2023). Moreover, existing commercial applications struggle to meet the needs of foot care and customized footwear. There is a need to improve software quality to support accurate measurements, enhance foot health awareness, and promote the prevention and treatment of foot problems (Kabir et al., 2021). 3D printing technology can provide personalized dressings and footwear solutions, improving fit and protection, which is beneficial for better managing DFU. Overall, 3D printing has great potential in DFU management, but development has been slow due to insufficient research and lack of precision in modelling for foot management. Therefore, we need a large number of preclinical and clinical studies to validate the benefits of 3D printing technology in DFU management.

## 8 Summary and outlook

**FIGURE 5:**
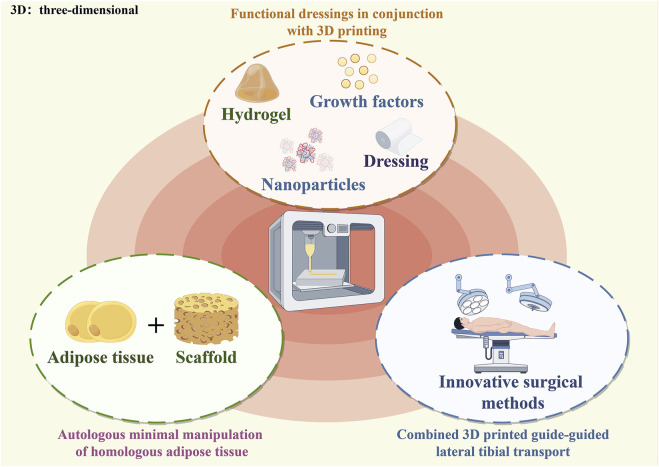
3D printing application in diabetic foot management.

3D printing technology offers unparalleled advantages, particularly in the realm of personalized treatment. The amalgamation of traditional treatment methods with 3D printing has yielded favorable outcomes in decelerating the progression of DFUs and facilitating wound healing. We summarize this in Figure 5. However, there is a limited body of research regarding the utilization of 3D printing technology in the domain of DFUs. The development of a 3D *in vitro* ulcer model that accurately simulates the hyperglycemic conditions *in vivo* is a significant challenge. One of the key factors in addressing the lack of 3D models is the promotion of keratogenic cell differentiation and proliferation. The reprogramming of iPSCs has also offered valuable insights for the construction of 3D skin models. Zebrafish share 87% genetic similarity with humans, pending development of experimental models close to humans. Effective 3D bioprinting dressings for chronic wounds are still lacking, in bi-layered biological dressings as well as dual AMPs are worth exploring by researchers. The development of wearable devices is still in its infancy. The development of sensors has to synergistically analyze the specificity of the skeletal and even the muscular structure of the foot, in addition to physical detection to better assess DFU diseases. There is a lack of awareness amongst medical professionals regarding the application of 3D printing technology in the management of DFUs.

## Data Availability

The original contributions presented in the study are included in the article/supplementary material, further inquiries can be directed to the corresponding authors.
